# Selective degradation processes in plants are not limited to autophagy

**DOI:** 10.1007/s00299-026-03806-9

**Published:** 2026-04-21

**Authors:** Kornel M. Michalak, Agnieszka Bagniewska-Zadworna

**Affiliations:** Department of General Botany, Institute of Experimental Biology, Faculty of Biology, Adam Mickiewicz University in Poznan, Uniwersytetu Poznańskiego 6, 61-614 Poznan, Poland

**Keywords:** Selective autophagy, Organelle removal, Degradation in plant cells, Programmed cell death, PCD

## Abstract

**Key message:**

**This review explores the various pathways of organelle degradation in plant cells. Besides autophagy, there are numerous other mechanisms for the selective removal of cellular components. Many of these mechanisms remain poorly understood, opening up new research avenues.**

**Abstract:**

Diverse degradation mechanisms of cellular components play a pivotal role in programmed cell death (PCD) during development and in response to stress. Notably, these degradation pathways can be genetically embedded in the differentiation of cells that, while remaining viable, adapt to specialized functions. Consequently, the heterogeneity of tissues and organs is shaped by intracellular destructions that lead to the autolysis of entire cells or merely a reduction of their cytoplasmic content. These degradation processes are integral to cell homeostasis, governing the requisite number of organelles and molecules through the removal of superfluous components. Autophagy is a critical process responsible for initiating and advancing this degradation. Selective autophagy meticulously identifies individual cell components destined for elimination. Despite the abundance of data regarding the functions and dynamics of this process at ultrastructural and molecular levels, the pathways involving varied degradation mechanisms in plant cells, which share common objectives, are exceedingly complex. These processes may interact with each other, be linked to autophagy, or operate independently. Furthermore, certain organelles may degenerate autonomously. Unlike selective autophagy, other degradation pathways in plant cells lack detailed descriptions. Specifically, the molecular regulation of various elimination pathways requires thorough documentation. Despite numerous discoveries through ultrastructural observations, significant gaps and uncertainties remain, necessitating further investigation. For plant cells, the descriptive collection and comparison of degradation processes have historically presented challenges, now addressed in this review.

## Introduction

The heterogeneity and functionality of plant tissues and organs are intricately linked to the framework of cell differentiation. Interestingly, during development, cell content may be both enriched and significantly reduced (Grafi 2004; Krishnamurthy et al. 2015). Intracellular degradation processes are pivotal for the progressive reduction of cytoplasmic content and may lead even to complete autolysis in cases of programmed cell death (PCD) (Jiang et al. 2021; Van Durme and Nowack 2016; Wang et al. 2024). Selective removal of specific cellular components occurs independently, yet diverse elimination pathways can also cooperate, underscoring the complexity involved in the destruction of cytoplasmic components (Kacprzyk et al. 2024; Schwarze et al. 2023), particularly when not culminating in cell death. These degradation processes are essential not only for restructuring, but also for maintaining cell viability. The complete degeneration of the protoplast in plants has been thoroughly documented. Such PCD mechanisms are prevalent in various developmental stages, including xylogenesis and senescence (Daneva et al. 2016; Kuriyama and Fukuda 2002; Van Hautegem et al. 2015). Similarly, numerous external factors trigger PCD in response to biotic and abiotic stresses (Ebeed and El-Helely 2021; Huysmans et al. 2017; Kabbage et al. 2017). However, degradation mechanisms also operate in cells, not destined for death, contributing to the establishment of functionality and homeostasis (Inoue et al. 2006; Otegui et al. 2024; Yano et al. 2007) as well as for removing damaged and redundant molecules or even entire organelles (Bassham 2007; Bosch and Franklin-Tong 2024; Marshall and Vierstra 2018; Schwarze et al. 2023). Furthermore, vacuole biogenesis relies on the delivery and digestion of such cytoplasmic cargo (Machado and Rodrigues 2019; Otegui et al. 2024; van Doorn et al. 2015). Products from these degradation processes are recycled to sustain cell viability (Avila-Ospina et al. 2014; Feng et al. 2015; Izumi et al. 2013). Moreover, despite being directed towards removal pathways, organelles undergo autonomous degeneration through internal reduction and complete collapse (Michalak et al. 2024a, b; Obata et al. 2011). Even after the initiation of programmed cell death (PCD) mechanisms, plant cells may remain viable until a critical threshold is reached (van Doorn 2005; van Doorn and Woltering 2005), a point dependent on the number of remaining mitochondria and plastids (Sobieszczuk-Nowicka et al. 2018). Precise initiation and management of restrained degeneration are required in cells that must remain viable. However, compared to bulk degradation, the cessation of mechanisms leading to cellular death or the specific removal of individual cell elements are not fully understood, particularly in processes other than those related to autophagy and vacuolar degradation. A comprehensive overview of similar or entirely distinct pathways of elimination targeting individual cell components will elucidate their roles in plants.

## Mechanisms and insights into selective autophagy

The primary process involved in this cellular degradation in plants is autophagy, distinguished into three types. In microautophagy, the tonoplast membrane invaginates, incorporating cytoplasmic fragments into the vacuole (Goto-Yamada et al. 2019; Li et al. 2012; Sienko et al. 2020). Macroautophagy is initiated by the formation of a membrane precursor that develops into a phagophore, which sequesters cytoplasmic cargo destined for degradation. The phagophore expands and eventually closes to form an autophagosome, a double-membrane vesicle. The autophagosome is subsequently delivered to the vacuole for digestion (Li and Vierstra 2012; Marshall and Vierstra 2018; Su et al. 2020; Yoshimoto et al. 2004). Mega-autophagy involves vacuole enlargement and tonoplast permeabilization, followed by disruption, leading to death through autolytic activity (Feng et al. 2022; Toyooka et al. 2023; van Doorn and Papini 2013; Wojciechowska et al. 2021). Considering the aspect of degradation selectivity, macroautophagy is recognized as the principal and most extensively characterized mechanism responsible for targeted removal of individual components of cytoplasm. Macroautophagic structures precisely recognize and bind to cell components destined for degradation (Floyd et al. 2012; Gatica et al. 2018; Marshall et al. 2022).

Selective autophagy (predominantly macroautophagy) has been recognized as a mechanism for stringent recruitment and progression of cellular destruction (Marshall and Vierstra 2018; Sienko et al. 2020). The regulation of this machinery involves autophagy-related proteins (ATGs), with the core ATG8, playing a crucial role at every stage of this pathway, conjugated to both membranes of autophagic structures (Nakatogawa et al. 2007; Xie et al. 2008). Although macroautophagy can act non-selectively, it also mediates selective cargo degradation when specific receptor proteins are involved. ATG8s, located on the inner membrane of the phagophore, drive such selective recruitment for degradation (Kellner et al. 2017) by binding to autophagy receptors as well as adaptors, and cargoes if they have autophagy-interacting motifs (AIMs). These motifs facilitate the connection between macroautophagic structures and selected cargo (Bu et al. 2020; Klionsky and Schulman 2014; Noda et al. 2010). Additional roles for ATG8s in selectivity were proposed, including preventing the fusion of preautophagic structures with different contents (Kellner et al. 2017; Maqbool et al. 2016) and the involvement of different ATG8 isoforms in PCD or selective autophagy (Michalak et al. 2024a, b). A functional specialization of ATG8 isoforms, mediating subcellular compartmentalization of differentially targeted autophagy pathways, has also been discussed (Stephani and Dagdas 2020). Generation of *A. thaliana* mutants lacking ATG8 isoforms has demonstrated their functional specialization across diverse cell types and stress conditions (Del Chiaro et al. 2025; Dong et al. 2025). Consequently, macroautophagy is considered a fundamental process in terms of degradation selectivity. For plant cells, autophagic receptors specific to cytoplasmic elements are well documented, and their mechanisms are extensively described in the literature. However, compared with yeast and mammalian systems, considerably less information is available for plants (Islam et al. 2019; Marshall and Vierstra 2018; Michaeli and Galili 2014; Ran et al. 2020; Rehman et al. 2021; Su et al. 2020). In contrast, microautophagy, involving the direct incorporation of cytoplasmic fragments into the vacuole through tonoplast invagination or protrusion (van Doorn and Papini 2013), traditionally has been considered a non-selective degradative process (Li et al. 2012). Nevertheless, emerging evidence suggests that specific organelles and cellular constituents can also be selectively sequestered through microautophagy. The molecular mechanism of selective microautophagy remains unclear; however, it can be an *ATG* gene-dependent process (Nakamura and Izumi 2019; Sienko et al. 2020). Anthocyanin aggregates that accumulate in close proximity to the vacuolar surface can be directly internalized by invagination of the tonoplast in a process resembling microautophagy. It has also been proposed that microautophagy mediates the vacuolar transport of other flavonoid aggregates, highlighting a previously underappreciated role of this pathway in secondary metabolite sequestration (Chanoca et al. 2015). Furthermore, microautophagy displays morphological similarities to the mechanism by which storage proteins are taken up into protein storage vacuoles (PSVs) in aleurone cells of the endosperm (Ding et al. 2022). Based on these structural and functional parallels, the cereal endosperm has been suggested as a suitable model tissue for investigating the molecular regulation of microautophagy in plants (Plott et al. 2025).

Autophagic mechanisms are undoubtedly of great importance, yet they are just one component of a broader system of selective removal processes. Many uncertainties persist regarding the reduction machinery activated in cells either locally-and-partially or against individual organelles. Ongoing research into plant autophagy is crucial, particularly given the plethora of known human selective autophagy receptors and their potential plant orthologs with as-yet-undefined functions (Stephani and Dagdas 2020). Nonetheless, significant gaps and misconceptions remain in our understanding of the entire selective degradation machinery, including autophagy-independent processes in plant cells. Comparative demonstrations of proven and potential mechanisms involved in other selective removal processes provide novel research directions. This review provides a detailed exploration of selective degradation processes, which have been somewhat overlooked, and whose mechanisms remain largely unknown compared to selective autophagy.

## Navigating the intriguing pathways of organelle degradation

**Fig. 1:**
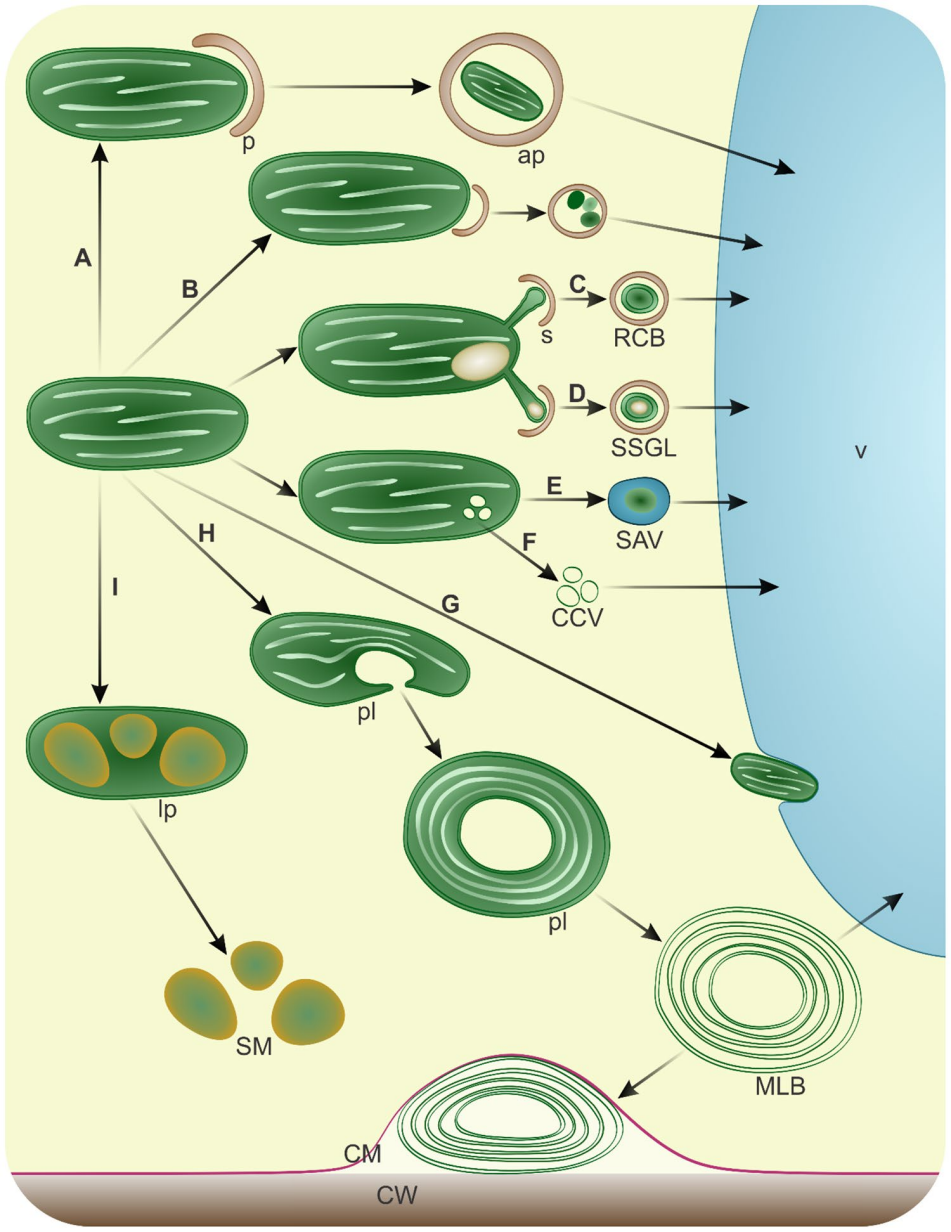
Pathways of possible plastid degradation in plant cells (note that figure is drawn up for all types of plastids, not only chloroplasts). **A** macroautophagy of whole plastid; **B** macroautophagy of plastid-derived components; *p*, phagophore; *ap*, autophagosome; **C** uptake of plastid fragment from stromule (s) resulting in the formation of rubisco-containing body (RCB); **D** uptake of plastid fragment from stromule (s) with starch grain resulting in the formation of small starch granule-like structures (SSGL); **E** Uptake of plastid fragment into senescence-associated vacuole (SAV); **F** formation and release of chloroplast vesiculation-containing vesicles (CCV); **G** microautophagy of plastid; **H** Formation of autophagic plastid-plastolysome (pl), which after degradation of inner content develops into multilamellar body (MLB) excreted out to the apoplast or to the vacuole; **I** leucoplast (lp) formation and break down to storage material (SM) release; *v* vacuole; *CM* cellular membrane; *CW* cell wall (not drawn to scale)

Plastid differentiation and homeostasis are contingent upon the degenerative processes affecting inner proteins and membranes (Lee et al. 2013), which are regulated by internal proteases (Choi et al. 2021; Jarvis and Lopez-Juez 2013; Nishimura et al. 2016; Osteryoung and Pyke 2014; Sakamoto 2006; Sierra et al. 2023) and autophagy as well (Wan and Ling 2022). The selective turnover of entire chloroplasts is known as chlorophagy or piecemeal chlorophagy for their fragments, though degradation is also crucial for other plastid types. Depending on the function or stimuli, plastids degrade through multiple pathways (Fig. 1). Macroautophagy is involved in the removal of entire plastids (Fig. 1A), such as photodamaged chloroplasts (Izumi et al. 2017). However, the recruitment of phagophores to chloroplast proteins (Fig. 1B) occurs during fruit ripening (Guo et al. 2025) and under conditions such as carbon starvation or salt stress (Michaeli et al. 2014). Plastid materials in autophagosomes can originate from stromules (Ni et al. 2022), resulting in the formation of rubisco-containing bodies (RCBs) (Fig. 1C) (Chiba et al. 2003; Ishida et al. 2008; Izumi et al. 2010; Spitzer et al. 2015) or small starch granule-like structures (SSGLs) (Fig. 1D) (Wang et al. 2013). The plastid envelope can also serve as one of the membrane sources for the formation of autophagy-related structures (Yanagisawa and Chuong 2023). Conversely, the removal of chloroplast materials is facilitated by lytic compartments such as senescence-associated vacuoles (SAVs) (Fig. 1E), specifically in senescing leaves as observed in *A. thaliana*, *Glycine max* (Otegui et al. 2005), and *Nicotiana tabacum* (Carrion et al. 2013; Martinez et al. 2008). Inner vesiculation within chloroplasts may be driven by the expression of the *CV* (chloroplast vesiculation) gene, leading to the formation and release of CV-containing vesicles (CCVs), which then fuse with the vacuole (Fig. 1F). This process is linked to leaf senescence or salt stress (Wang and Blumwald 2014). Similar vesicles have been observed around the periphery of the plastid or forming a cluster in plastids of floral nectaries in *Geranium phaeum* (Konarska and Masierowska 2020). The uptake of SAVs and CCVs by vacuoles remains elusive (Otegui 2018); it has been proposed to define this process as microchlorophagy (Zhuang and Jiang 2019), but simple fusion with the tonoplast should also be considered. Direct plastid incorporation by the vacuole (Fig. 1G) has been detected in chloroplasts accumulating reactive oxygen species (Fisher et al. 2022; Lemke et al. 2021; Woodson et al. 2015) or after induced expression of the synthetic chlorophagy receptor (Liu et al. 2025). These organelles can also degrade autonomously, curling to engulf a portion of the cytoplasm and developing into plastolysomes (Fig. 1H). In senescing embryo-suspensor cells, autophagic activity of plastolysomes has been detected by the localization of acid phosphatase in *Phaseolus coccineus* (Nagl 1977), with further confirmation in *Ph. vulgaris* and *Tropaeolum majus* (Gartner and Nagl 1980). In *Brassica napus* embryogenic microspores, these autophagic plastids transform into multilamellar bodies (MLBs) after degradation including incorporated cytoplasm. Membrane remnants of plastolysome-derived MLBs are excreted from the protoplast to the apoplast (Parra-Vega et al. 2015). The formation and degradation of these structures are scarcely known and may relate to autophagy or vacuole-independent removal. Numerous plastolysomes and MLBs in vacuoles have been observed during embryogenesis in *Picea abies* (Filonova et al. 2000). In senescing petals of *Dendrobium* cultivar, plastolysomes and MLBs in vacuoles were observed concurrently with microautophagy and macroautophagy (van Doorn et al. 2015). Similar degeneration of plastids ending with transformation into irregular MLBs was noted in the xylem parenchyma in stems of *Pinus sylvestris* (Saranpää 1988) and in *Nannochloropsis oceanica* during nitrogen starvation (Roncaglia et al. 2021). In photosynthetic tissues, the formation of plastolysomes from chloroplasts has not been reported. However, cup-shaped plastids with thylakoid-like linear structures enclosing cytoplasmic portions have been observed in glandular parenchyma cells during floral nectary development in *Geranium phaeum* (Konarska and Masierowska 2020). Swelling, the appearance of membrane-free areas, and disorganization of the thylakoid system leading to MLB-like plastids have been observed in *Picea abies* needles during seasonal changes (Senser et al. 1975). Under conditions of nitrogen and phosphorus deprivation after the breakdown of chloroplast glycerolipids numerous MLBs attached to the tonoplast or within autophagic vacuoles have been seen in *Lobosphaera incisa*. These stress conditions enhance autophagy and lead to the formation of lipid droplets (Kokabi et al. 2020). The transition of other plastid types from pre-existing chloroplasts also involves extensive internal degradation processes. During fruit ripening in *Solanum lycopersicum*, proteolytic pathways contribute to chromoplast differentiation by facilitating the removal of chloroplast-specific components (Ling et al. 2021). Recent evidence indicates that this remodeling process is mediated by autophagy. Furthermore, autophagy has also been implicated in the degradation of carotenoid-containing chromoplasts at late stages of fruit ripening and has been termed chromophagy (Guo et al. 2025). Plastids may also degrade completely, allowing cells direct access to synthesized compounds (Fig. 1I). In some differentiated cells, plastid-derived reserve materials are not confined by a membrane (Fan et al. 2019; Goossens et al. 1988; Nakayama et al. 2012; Sanjbod et al. 2023; Tratt et al. 2009; Wei et al. 2008). In the event of sudden demand and deficiency, materials in plant cells can be provided by recycled macromolecules of plastid degradation (Havé et al. 2019). It is a common stress response (Woodson 2022); during starvation, plastids degenerate, releasing nutrients that enable survival and the weathering of unfavorable periods, instead of causing total cellular damage (Avin-Wittenberg et al. 2015; Siqueira et al. 2018). Removal pathways are also activated against damaged chloroplasts (Izumi et al. 2017, 2010; Izumi and Nakamura 2017) or unnecessary plastids in sperm cells (Primavesi et al. 2017). It is unclear whether the degradation processes mentioned are applicable to all types of plastids and may be involved in the development of vascular tissues containing non-photosynthetic plastids. However, under conditions of high light intensity, chloroplasts are present in xylem parenchyma, as well as in all types of undifferentiated xylem cells (Burrows and Connor 2020; Cocoletzi et al. 2013). Similarly, numerous chloroplasts are observed in tracheary elements of *Zinnia elegans* cultivated in vitro (Obara et al. 2001). They lose photosynthetic activity (Novo-Uzal et al. 2013) and are presumably removed through an unknown pathway. Moreover, the gene encoding CCV exhibited increased expression in the *A. thaliana* root, mostly in the endodermis, but also in the developing vascular stele (Ryu et al. 2019). The number of plastids also decreases during the development of sieve elements (Evert 2006), and they are transited into starch grains (Knoblauch and van Bel 1998; Palevitz and Newcomb 1970). However, before sieve element maturation, some of them are removed by the formation of plastolysomes. Derived MLBs fuse with autophagosomes, are observed in vacuoles and are excreted to the apoplast (Michalak et al. 2024a, b). Similar plastid forms were described as amoeboid or cup-shaped during xylem differentiation of *Hordeum vulgare* roots (Lux 1986).

**Fig. 2:**
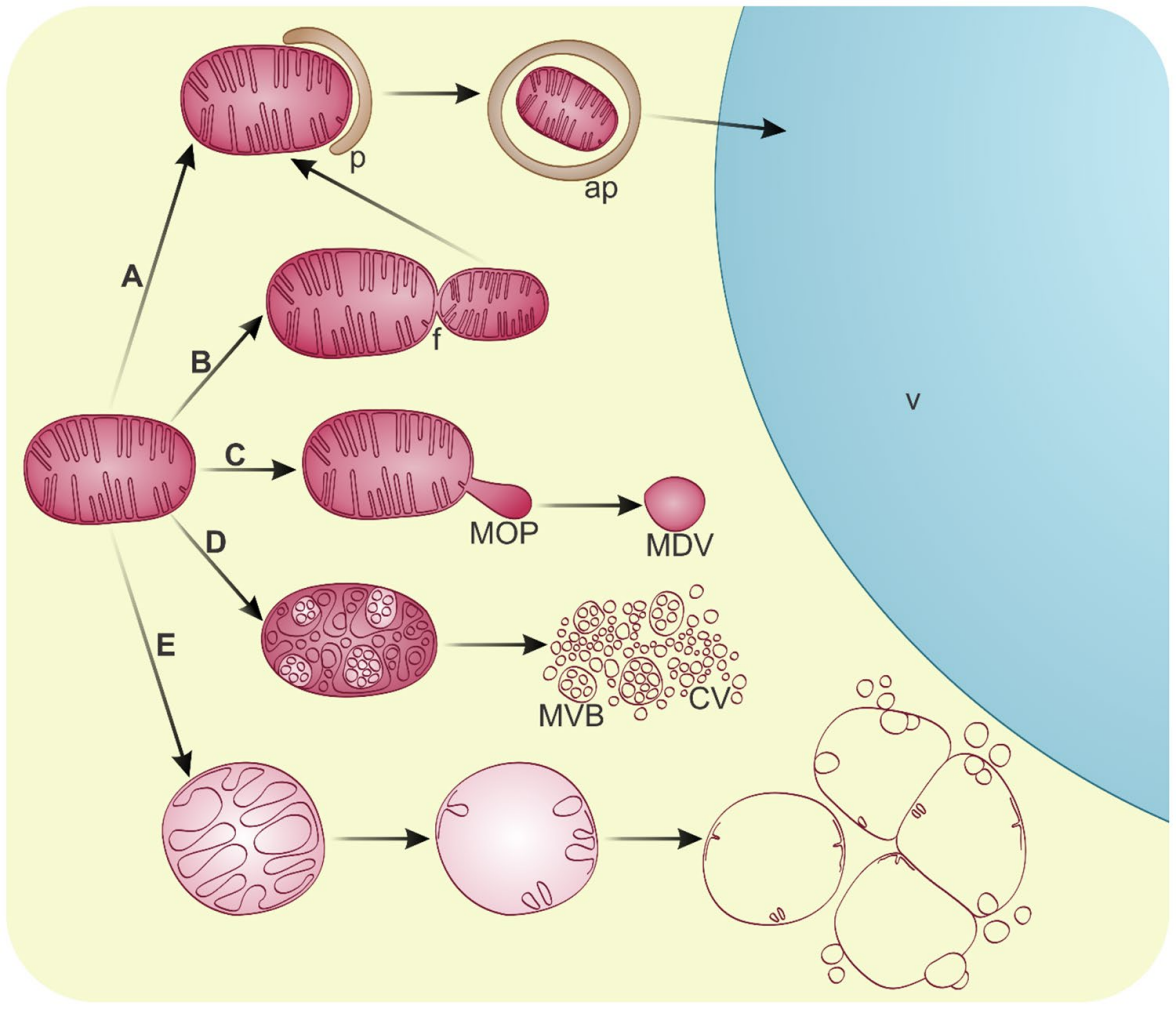
Degradation of mitochondria in plant cells. **A** macroautophagy of mitochondrion; *p* phagophore; *ap* autophagosome; *v* vacuole; **B** mitochondrial fission (f) preceding the macroautophagy pathway; **C** mitochondrial outer-membrane protrusion (MOP) and formation of mitochondria-derived vesicle (MDV); **D** inner vesicle formation and proliferation of the outer mitochondrial envelope resulting in the generation of multivesicular bodies (MVB) and cytoplasmic vesiculation (CV); **E** mitochondrion swelling with reduction of the matrix and inner membranes resulting in the formation of vesicles with cristae remnants, which are able to fuse or to proliferate the outer membrane (not drawn to scale)

Mitochondrial biogenesis and functionality are closely linked to the proteolytic degradation processes of inner proteins (Heidorn-Czarna et al. 2022; van Wijk 2015). The progression of programmed cell death (PCD) largely depends on transitions in mitochondrial morphology and metabolomics. Both disrupted and excessively activated mitochondrial production of reactive oxygen species (ROS) significantly impact cellular destruction. Moreover, certain mitochondrial proteins act as either positive or negative regulators of PCD (Moller et al. 2021), making changes in mitochondrial quantity critical in cytoplasmic degradation. During leaf senescence, while chloroplasts are the first to degrade, mitochondria are the last (Keech et al. 2007). Mitochondria orchestrate the catabolic processes that are involved in the remobilization of nutrients derived from the degradation of other cellular components (Chrobok et al. 2016). However, selective degradation processes of mitochondria are significantly less documented compared to those of plastids (Fig. 2). Dynamic morphological alterations of mitochondria have been observed under stress conditions that lead to cell death (Yoshinaga et al. 2005) and during senescence (Zottini et al. 2006). The removal of entire mitochondria through macroautophagy (mitophagy) (Fig. 2A) facilitates the elimination of depolarized or aggregated mitochondria, thus aiding in the nutrient remobilization (Nakamura et al. 2021a, b). Additionally, mitophagy plays a crucial role in maintaining energy homeostasis and protecting against mitochondrial dysfunction (Li et al. 2022a, b). In leaves of *A. thaliana* during UVB damage, autophagy contributes to mitochondrial quality control (Nakamura et al. 2021a, b). Despite being damaged, redundant mitochondria after fission are also removed via autophagy (Fig. 2B) in plant cells, such as during spermiogenesis in *Marchantia polymorpha* (Norizuki et al. 2022). Hence, it is possible to eliminate only fragments without destroying the entire organelle. Mitochondrial outer-membrane protrusions (MOPs) and mitochondria-derived vesicles (MDVs) (Fig. 2C) have been documented in leaves of *A. thaliana* during dark-induced senescence. It has been proposed that MDVs, similar to plastid-derived RCBs, can be mobilized to the vacuole via autophagy (Yamashita et al. 2016). The removal of entire mitochondria, as well as MDVs, has been confirmed as autophagy-related for mammals (Pan et al. 2023) and mitochondrial-derived compartments (MDCs) for yeast (Malmgren Hill and Nystrom 2016). Other degradation processes of mitochondria in plants include inner vesicle formation and proliferation of the outer mitochondrial envelope, resulting in the generation of multivesicular bodies (MVBs) and cytoplasmic vesiculation (Fig. 2D). Such mitochondrial disintegration has been observed in plants infected by viruses (Hatta and Ushiyama 1973; Rubino et al. 2014). During reduced photosynthesis, mitochondria become abnormally elongated and lack cristae and matrix (Fukushima et al. 2022). In *Nitella flexilis* mitochondria swell and show a decrease in matrix density under extremely low light intensity (Foissner 1983). This swelling and dysfunctionality are triggered by prolonged opening of the mitochondrial permeability transition pore (MPTP). However, similar mitochondrial degeneration has been documented during developmental processes. During sieve element differentiation, mitochondria swell along with a reduction of matrix density and inner membranes, resulting in the formation of vesicles with cristae remnants. They also exhibit outer membrane proliferation and subsequently fuse (Michalak et al. 2024a, b). This selective mitochondrial degradation (Fig. 2E) occurs even though they are present in low numbers in mature sieve elements (Evert 2006; Froelich et al. 2011). Conversely, in developing tracheary elements of *Zinnia elegans*, just before vacuolar collapse, the structure of mitochondria clearly alters. Initially, swollen cristae are observed, followed by depolarization and disintegration of the outer membrane (Yu et al. 2002).

**Fig. 3:**
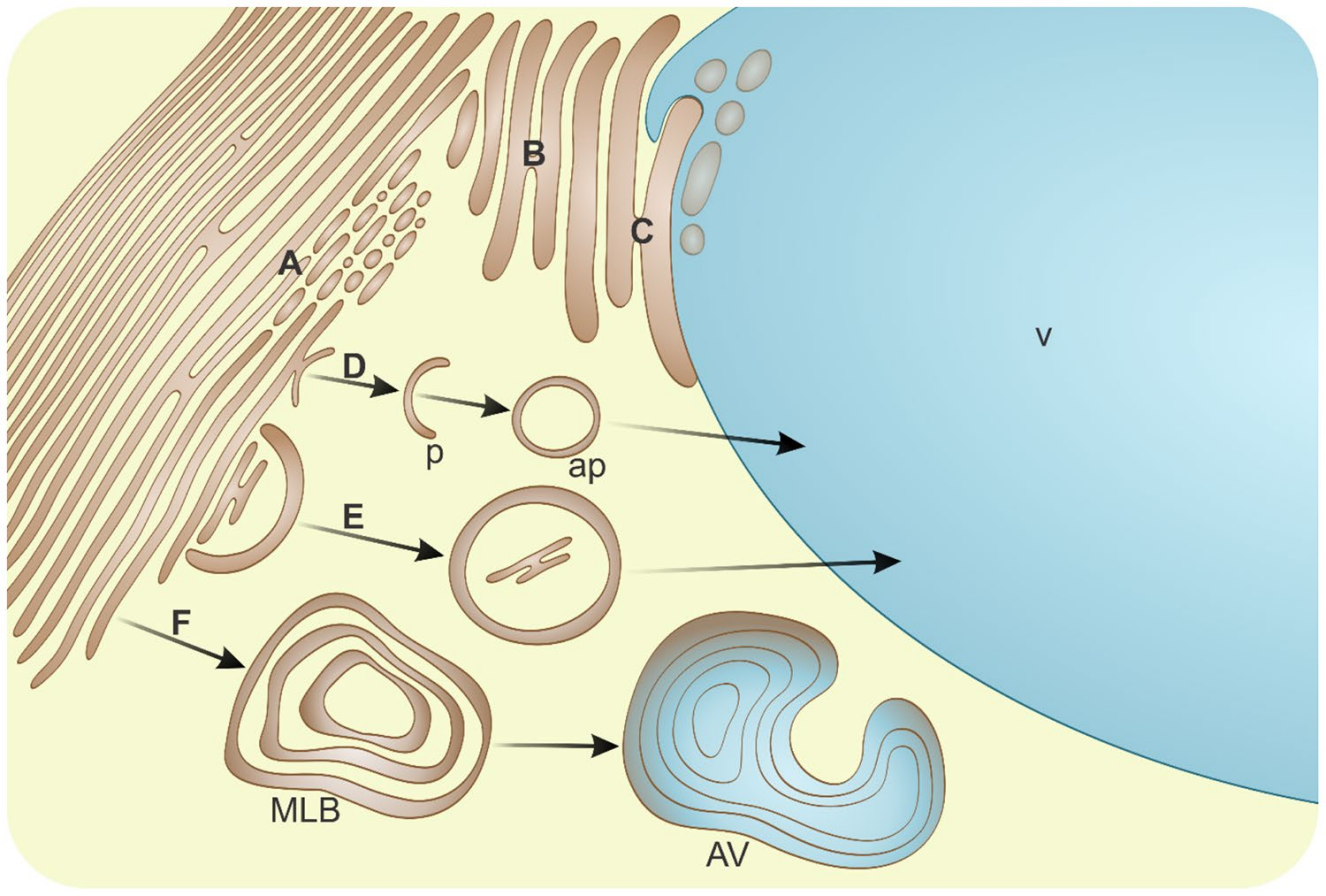
Pathways of endoplasmic reticulum degradation in plant cells. **A** ER sheet fragmentation into tubules, cisternae or vesicles; **B** changing into swollen inclusions via dilation; **C** compression of dilated ER to adjacent vacuoles and fusion with tonoplast; **D** formation of ER-derived macroautophagic structures; *p* phagophore; *ap* autophagosome; **E** macroautophagy of ER; **F** formation of concentric ER similar to multilamellar body (MLB) and development into autophagic vacuole (AV); *v* vacuole (not drawn to scale)

The endoplasmic reticulum (ER) in plant cells is vital for biosynthesis, and the homeostasis of various compounds is crucial for organellar compartmentalization. Moreover, the ER is interconnected with the nuclear envelope, membranes of other organelles, the cytoskeleton, and through plasmodesmata to adjacent cells (Brandizzi 2021; Kriechbaumer and Brandizzi 2020; Okita and Rogers 1996). To fulfill such roles, the ER-derived membrane system dynamically and continuously adapts (Pain et al. 2019) or repositions (Sparkes et al. 2009) via various processes (Fig. 3) influenced by both internal and external factors. This network of remodelling includes the formation of long, regular sheets and also encompasses divisions and transformations into smaller, interconvertible structures (Gupton et al. 2006; Kriechbaumer et al. 2018; Li et al. 2022a, b; Sparkes et al. 2010; Tolley et al. 2008), such as tubules, cisternae and vesicles (punctae) (Fig. 3A). The appearance of packed and longitudinally oriented ER can shift through dilatation (Fig. 3B) into swollen inclusions (Bones et al. 1989). In roots of various *Brassicaceae* species, the number of dilated ER cisternae increases with differentiation. While similar structures are present in leaves and stems, they are significantly less common in *Papaveraceae* and *Resedaceae* (Iversen 1970). Nonetheless, ultrastructural analyses have shown that the presence of dilated ER is typical across different organs, tissues, and cell types in many species, albeit these structures can be long and utricular or irregularly shaped (Behnke and Eschlbeck 1978). Such structures have been identified as ER bodies in *Brassicaceae,* formed as chemical defense containing endogenous substrates that deter herbivores (Yamada et al. 2020). However, in *A. thaliana*, extensive dilations have been linked with loss of ER functionality, following PCD induced by fungal infections (Liu et al. 2023). Dilated ER participates in lytic vacuole biogenesis (Fig. 3C) (Okita and Rogers 1996; Viotti et al. 2013). In maturing root cells of *Avena sativa*, extensive ER dilation leads to compression of adjacent vacuoles and fusion with the tonoplast (Herman et al., 1994). Additionally, ER membranes are the origin of autophagic structures (Fig. 3D) (Luo et al. 2023; Zhuang et al. 2017). Consequently, macroautophagy is a process regulated by ER (Morel 2020; Zhuang et al. 2018) and is responsible for the selective removal of its own fragments (Fig. 3E) (Bao and Bassham 2020; Liu and Bassham 2013; Zeng et al. 2019). Specific ER-phagy (or reticulophagy) receptors differentiate between sheets or tubules (Beese et al. 2019). To maintain physiological balance, surplus membranes and aberrant polypeptides of the ER are also removed by micro- and macroautophagy (Li et al. 2023; Molinari 2021; Rudinskiy and Molinari 2023). Hence, degradation mechanisms are integral both in the organization and regression of ER structure. Lamellar or tubular aggregates have been observed in leaves of *Triticum aestivum* infected by viruses (Xie et al. 2019). Following the initiation of degradation mechanisms under stress conditions, the ER morphs into various shapes, forming complicated multilamellar bodies (MLBs). During PCD induced by tungsten in root tip cells of *Pisum sativum*, MLBs arise from concentric accumulation of ER membranes enclosing cytoplasm fragments (Adamakis and Eleftheriou 2019). A similar spiral membranous structure of the cytoplasm was noted within the vacuole during PCD in senescing petals of *Antirrhinum majus* (Nabipour Sanjbod et al. 2023). In *Lilium* pollen, the development of concentric ER saccules into autophagic vacuoles has been documented (Fig. 3F) (Pacini et al. 2011). Proteins that drive membrane fusion, curvature, and tubule stabilization as well as ER-phagy receptors are specialized shapers of the ER (Brandizzi 2021; Stephani et al. 2020; Yang et al. 2021). The specific elimination of accumulated proteins related to biotic and abiotic stresses is also essential to restore ER homeostasis. This response is facilitated by components of ER-associated degradation (ERAD) (Duan et al. 2023; Strasser 2018; Vembar and Brodsky 2008), which recognize misfolded proteins and direct them from the ER to the cytosol to proteasomes or autophagy pathways (Chen et al. 2020). One of the PCD events during xylogenesis is the formation of elongated vesicles from swollen ER cisternae (Bagniewska-Zadworna et al. 2012). Thus, dilated ER as an unnecessary structure is a potential target for intracellular degradation. It is removed by microautophagy-like vacuolar uptake both in xylem (Esau 1975) and phloem (Michalak et al. 2024a, b).

**Fig. 4:**
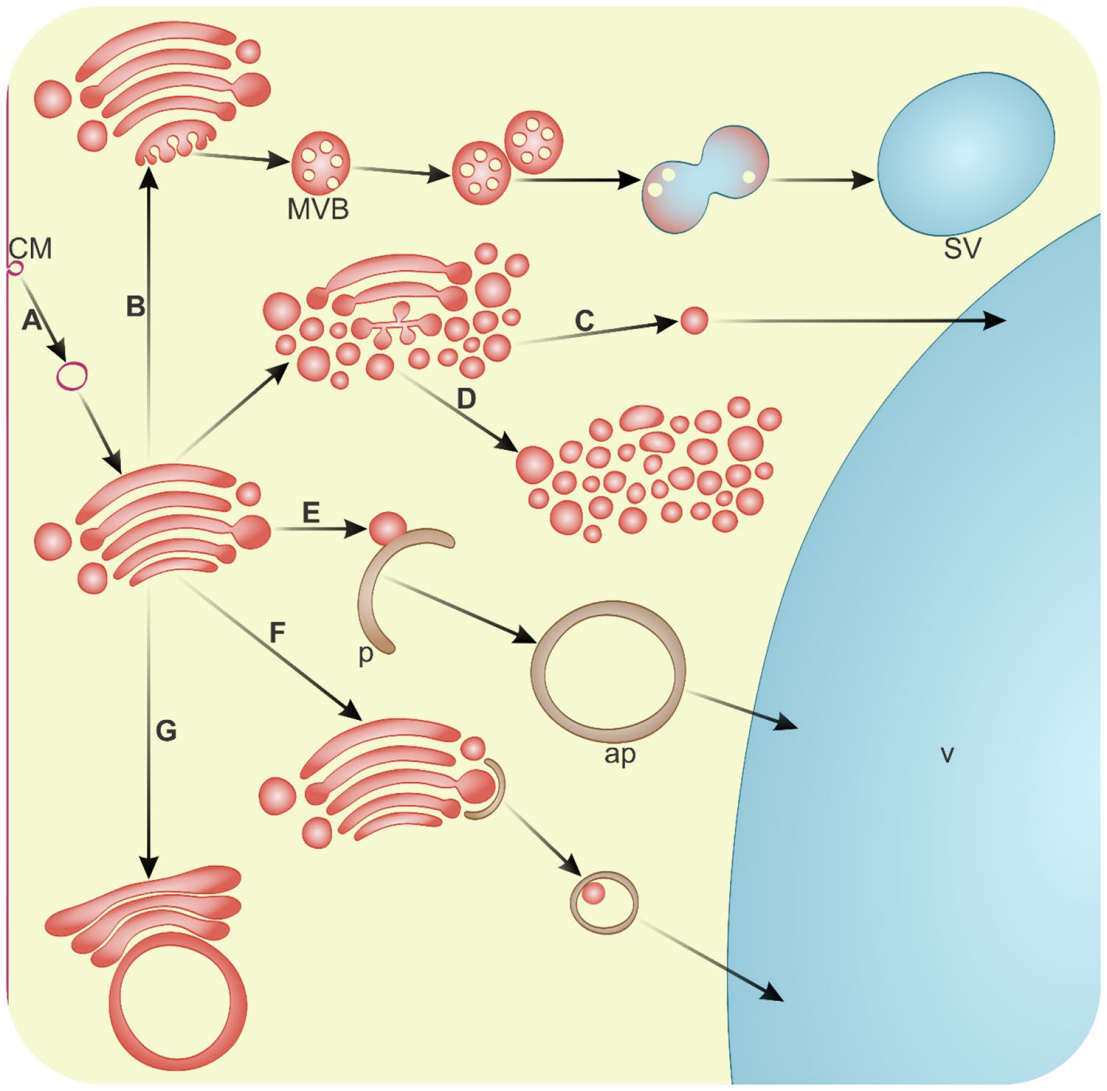
Pathways of Golgi apparatus degradation in plant cells. **A** GA-dependent cellular membrane (CM) degradation; **B** GA cisternae disintegration into multivesicular bodies (MVBs), which after fusion form small vacuole (SV); **C** acceleration of vesicle trafficking with lytic compounds; **D** dictyosome fragmentation and extensive vesiculation; **E** involvement of GA-derived vesicles in the progression of macroautophagic structures; *p* phagophore; *ap* autophagosome; **F** macroautophagy of GA; **G** swelling of GA cisternae and development into autophagosome-like vesicles (not drawn to scale)

The Golgi Apparatus (GA) in plant cells, comprising membrane systems of cisternae stacks (dictyosomes) and vesicles, is pivotal for sorting and secreting various cellular cargoes (Hawes and Satiat-Jeunemaitre 2005; Robinson 2020). The GA may degrade itself (Fig. 4) but plays also an important role in the regulation of programmed cell death (PCD) mechanisms (Liu et al. 2023), and in the removal of membranes (Fig. 4A) within viable cells (Schwihla and Korbei 2020; Zhang and Hicks 2019). In non-growing secretory cells of *Z. mays* root cap excess membranes are removed by GA vesicles (Mollenhauer et al. 1991). The delivery of these GA vesicles to vacuoles is crucial for determining their functions (Goring and Di Sansebastiano 2017; Hawes 2005; Nebenfuhr and Staehelin 2001; Paez Valencia et al. 2016; Shimizu et al. 2021), with enzymatic digestion in lytic vacuoles dependent on GA sorting (Jiang and Rogers 2018; Robinson et al. 2008; Scheuring et al. 2012). GA cisternae, depending on the cell cycle, disintegrate into multivesicular bodies (MVBs), which upon fusion initiate vacuole formation (Fig. 4B). This biogenesis of vacuoles is reliant on GA-delivered MVBs, a process validated in differentiating cortex cells of *A. thaliana* root (Cui et al. 2019). PCD mechanisms are characterized by extensive vacuolation and the enlargement of the central vacuole (van Doorn et al. 2011). In shoot apical meristem and leaf primordia of *Arabidopsis thaliana*, the total volume of MVBs increases (Segui-Simarro and Staehelin 2006). Observations in root columella cells of *A. thaliana* (Kang et al. 2011) and basal endosperm transfer cells of *Zea mays* have shown GA cisternae shrinkage and fragmentation into vesicles (Kang 2011; Kang et al. 2009). Significantly, an increased number of these GA-derived structures is associated with accelerated trafficking (Fig. 4C), a symptom of PCD, culminating in GA being consumed by vesicle production (Fig. 4D) (Cacas 2010). The GA is instrumental in autophagy, delivering vesicles containing membranes and lipids necessary for the progression of preautophagic structures (PAS) (Fig. 4E) and vacuole biogenesis (Chan and Tang 2013; Filonova et al. 2000; Li and Vierstra 2012; Zeng et al. 2021; Zhuang et al. 2017), as well as enzymes, enabling degradation to commence within autophagosome before their delivery to the vacuole (van Doorn and Papini 2013). In animals, GA recruitment by macroautophagic structures is well documented (Chang and Yang 2022; Lu et al. 2020; Nthiga et al. 2021a, b; Rahman et al. 2022), including a common autophagy receptor with the ER (Nthiga et al. 2021a, b). Plants also possess ATG8 isoforms potentially interacting with specific GA membrane receptors (Fig. 4F), including those related to MVBs (Zeng et al. 2021), although this interaction has not yet been confirmed *in planta*. ATG8 is involved in the recovery of dilated membranes to functional GA, a process delayed in the *atg5* mutant; however, this function of ATG proteins has been described as independent of autophagy (Zhou et al. 2023). The full extent of autophagic structures’ involvement in GA degradation in plant cells remains unverified. During the senescence of *Antirrhinum majus* petals, an irregular organization of the GA with fewer cisternae and no vesicles has been observed. Additionally, the formation of multimembrane autophagosome-like structures (Fig. 4G) arising from residual GA cisternae undergoing degradation through swelling and fusion has been documented (Nabipour Sanjbod et al. 2023). In yeast and animals, entire transformed cisternae can serve as a membrane source for phagophores (Yamaguchi et al. 2016). In *Populus trichocarpa* roots during PCD of xylem, accumulations of GA with a low number of cisternae compared to a high number of vesicles have been observed (Bagniewska-Zadworna et al. 2012). This occurrence of structures and MVBs was monitored during selective cytoplasmic degradation throughout phloemogenesis (Michalak et al. 2024a, b).

**Fig. 5:**
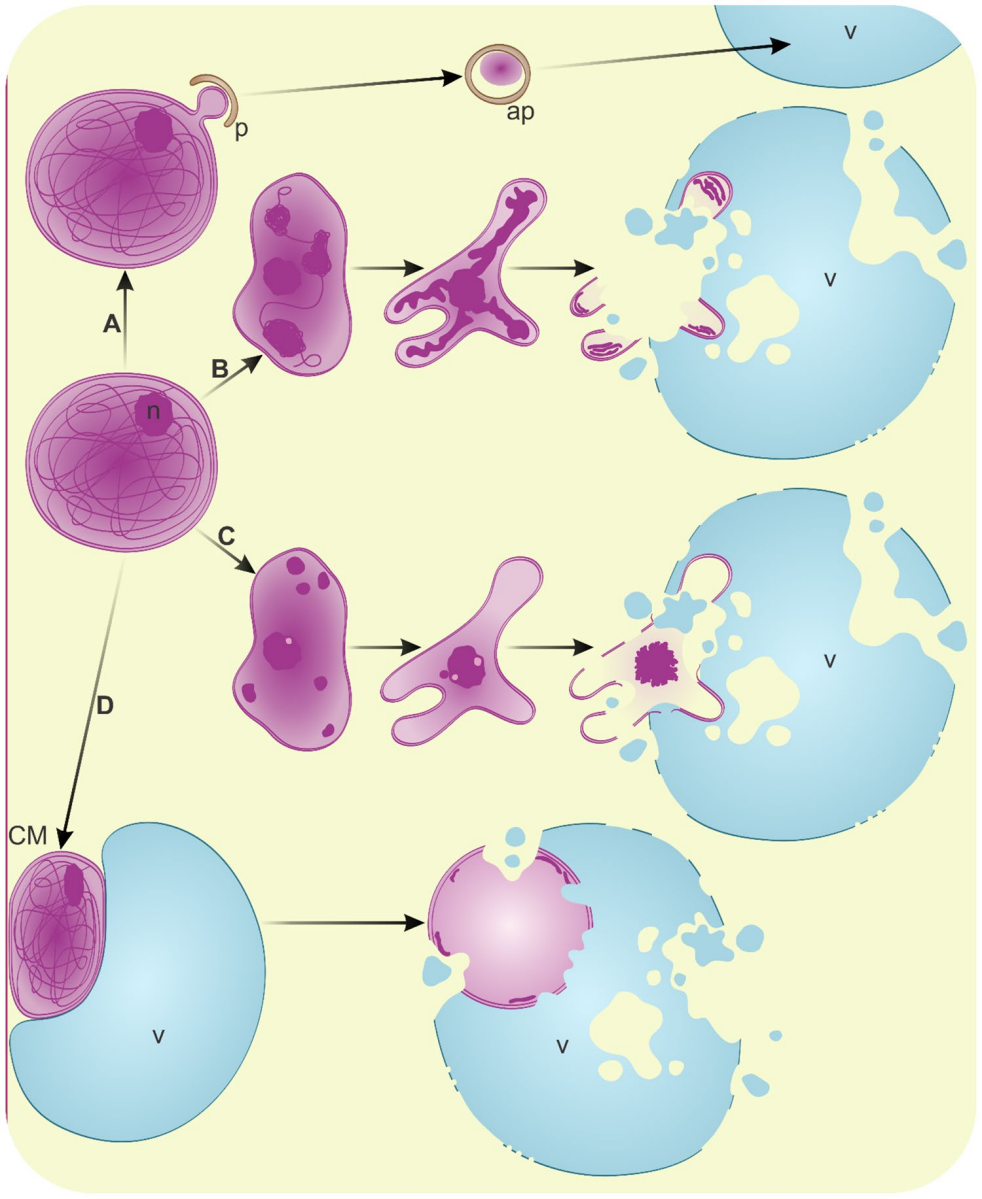
Pathways of nucleus degradation in plant cells. **A** nucleus macroautophagy; *p* phagophore; *ap* autophagosome; **B** chromatin condensation and degradation of abnormally shaped nucleus after mega-autophagy; **C** local changes in nucleus content density and nucleus disintegration followed by nuclear envelope permeabilization and nucleolus breakdown after mega-autophagy; **D** lobed nucleus compressed between cellular membrane and vacuole and its return to spherical structure and degradation after mega-autophagy; *n* nucleolus; *v* vacuole (not drawn to scale)

A prominent feature in plant cells undergoing programmed cell death (PCD) is nucleus degradation (Fig. 5), despite extensive vacuole-related destruction (van Doorn et al. 2011) marking non-reversible death (van Doorn and Woltering 2004). So far, nuclear autophagy (or nucleophagy) (Fig. 5A) in plants has been identified as a response to viral infection (Li et al. 2020). However in most cases, selective degradation of the nucleus results in complete enucleation. Caspases cleave nuclear proteins and facilitate the detachment of the nuclear envelope from chromatin. This separation allows access for factors that drive chromatin condensation and fragmentation of nuclear DNA (Latrasse et al. 2016; Petrovská et al. 2015). In the final stages of PCD during embryogenesis in *Picea abies*, before vacuole collapse and protoplast autolysis, the nucleus is lobed and subsequently segmented (Filonova et al. 2000; Smertenko et al. 2003). In leaf senescence of *Brassica napus*, post-chromatin condensation, the nucleus is fragmented (Fig. 5B) (Watanabe et al. 2002). This process is associated with nuclease activity as confirmed in *A. thaliana* (Farage-Barhom et al. 2011). Conversely, in microspores of plants with cytoplasmic male sterility and tapetal cells, chromatin rearrangements are less pronounced; changes in plasma and nucleolus have been noted. The final degradation involves the disintegration and dispersal of the nuclear envelope, leading to detached nucleolus (Fig. 5C) (González-Melendi et al. 2008). There is considerable variability in nuclear degradation in plant cells; even within the same cell type, such as sieve elements which undergo specific enucleation, this process can vary significantly by species. In *Triticum aestivum* caryopsis, chromatin is typically condensed, but its volume decreases and the envelope becomes obscure at the end of sieve element differentiation (Wang et al. 2008). In the roots of *A. thaliana*, a progressive decrease in nuclear content, irregular structure, and breakdown are observed in developing sieve elements (Furuta et al. 2014). Similarly, in sieve elements of *Populus trichocarpa* roots, minimal changes in the density of nuclear plasma and nucleolus are noted compared to the extensive decrease in nuclear content at the end of sieve element differentiation (Michalak et al. 2024a, b). These changes are associated with alterations in nuclear structure, such as the formation of blebs or invaginations (Fig. 5D). Subsequently, the envelope becomes sparse and fragmented, along with the nucleolus becoming detached (Michalak et al. 2024a, b). On the other hand, during in vitro differentiation of tracheary elements of *Zinnia elegans*, the lobed nucleus is reshaped by the central vacuole. The tonoplast expands pressing the nucleus against the plasma membrane. Following vacuole rupture, in this case, the nucleus becomes spherical without any folding and soon completely degrades (Fig. 5D) (Obara et al. 2001), a process linked to the activity of DNases and nucleases (Ito and Fukuda 2002). However, in roots of *Populus trichocarpa* characteristic chromatin condensation was not observed in developing tracheary elements (Bagniewska-Zadworna et al. 2014).

## Conclusions and research perspectives

Selective autophagy plays a pivotal role in cellular homeostasis by targeting specific organelles and molecules for degradation. This process is especially crucial in plants, where it supports both developmental processes and stress responses. Despite noteworthy advances in this field, significant knowledge gaps persist in our understanding of selective autophagy in plants, particularly in the areas of golgiphagy and nucleophagy. Research has acknowledged the significance of the ATG8-interactome (Zeng et al. 2021) and established a link between autophagy and the organization of the Golgi apparatus (GA) (Zhou et al. 2023). However, definitive evidence for GA recruitment by autophagic structures remains elusive. While detailed studies in mammals have described autophagic receptors that target the GA in the process of golgiphagy (Hickey et al. 2023), analogous mechanisms in plant cells have not yet been clearly identified. Nucleophagy, particularly during viral infections, has been observed in various plant cell types (Li et al. 2020). This contrasts with mammalian cells, where autophagosomes can originate directly from the nuclear envelope to encapsulate nuclear fragments (Chen et al. 2014). This distinction underscores the need for further research to uncover novel insights into plant-specific autophagy pathways. Interestingly, in plant cells, plastids can function similarly to autophagosomes, termed plastolysomes. The dynamics of these specialized plastids have been explored through ultrastructural studies and three-dimensional visualization techniques (Parra-Vega et al. 2015). However, the specific genes responsible for this autophagic mechanism have not been identified. Notably, similar curled plastids, observed in the xylem and previously referred to as ‘amoeboid plastids’ due to their amoeba-like structure, have been documented (Lux 1986). Following bacterial digestion, amoebae release multilamellar bodies (Karas et al. 2023; Paquet et al. 2013), which are comparable or possibly analogous to those from plastolysomes in plant cells.

Novel experimental designs are required to more effectively explore the similarities and differences between PCD mechanisms and selective degradation processes in plants. One of the models for studying the genetically programmed removal mechanisms of cell components is the differentiation of vascular tissues. The plant vasculature is a complex system comprising several cell types that share a common origin (Scarpella and Meijer 2004). In the xylem, conductive cells known as tracheary elements (TEs) at maturity are dead and devoid of contents, facilitating the transport of water and minerals. Conversely, in the phloem, sugar distribution is managed by sieve elements (SEs), which remain viable post-differentiation despite enucleation and substantial cytoplasmic reduction. This adaptation to substance transport culminates in the complete degradation of cytoplasmic content in TEs and its depletion in SEs, respectively (Lucas et al. 2013). All three types of autophagy were documented during xylogenesis. Microautophagy and macroautophagy are responsible for progressing cytoplasmic clearance (Bagniewska-Zadworna et al. 2012; Kwon et al. 2010, 2011; Wojciechowska et al. 2019). Mega-autophagy is the last stage of PCD, ending with the emptying of the TEs through autolytic activity (van Doorn and Papini 2013). However, evidence for selective autophagy during xylogenesis is less prevalent compared to autophagic mechanisms associated with vacuole rupture and subsequent protoplast collapse (Bagniewska-Zadworna et al. 2012; Wojciechowska et al. 2019). In roots of *Arabidopsis thaliana*, the breakdown of the central vacuole is preceded by the accumulation of enzymes, such as cysteine proteases (Avci et al. 2008; Escamez et al. 2016). In stems of *Populus deltoides* the integrity of the tonoplast is transient, quickly followed by autolysis within the vessel elements (Yin and Fan 2009), unlike the prolonged selective autolysis observed in SEs (Michalak et al. 2024a, b; Yin and Fan 2008). In developing SEs of *Triticum aestivum* caryopsis, coincident microautophagy and vacuole enlargement with tonoplast disintegration were observed (Wang et al. 2008; Yang et al. 2015), alongside documented protease activity and protoplast acidification (Yang et al. 2015). However, such processes were not evident during cytoplasmic degradation in SEs of *A. thaliana* roots (Furuta et al. 2014). Macroautophagy is proposed as a critical selective degradation process in SE differentiation, contrasting with the involvement in PCD in TEs (Wojciechowska et al. 2021), and this occurrence of macroautophagy, confirmed by ATG8 localization, is common across different species (Michalak et al. 2024a, b). There are numerous selective autophagy pathways controlled by ATG8 isoforms and autophagic receptors. Concurrently, macroautophagy and microautophagy can remove protoplast elements, participating in the biogenesis of lytic vacuoles, which upon expansion, can perform complete protoplast lysis via mega-autophagy. These processes collectively initiate a degradation machinery mediated by various factors, determining whether a cell is directed towards PCD, as in TEs or towards partial depletion, as in SEs. This machinery is notably complex, as it encompasses numerous processes that culminate in degradation. Detailed analyses have revealed that selective autophagy is essential for SE content depletion, but it is accompanied by numerous other processes. Conversely, the degradation of lytic vacuoles plays a key role in reducing cytoplasm density and occurs locally at the onset of SE differentiation. In the final stages of phloemogenesis, the cell volume is predominantly filled with low-density cytoplasm (Michalak et al. 2024a, b). The removal mechanisms leading to this state, but not to cell death, are adaptations for substance transport through the lumen of the sieve tube. Monitoring xylem and phloem from meristematic tissue to specialized cells reveals diverse degradation modalities. Understanding what initiates, regulates and potentially halts degradation is equally crucial, yet remains poorly understood. Characterizing these pathways will help identify the factors determining whether degradation simply removes cytoplasm or advances due to cell functionality.

Understanding the intricate mechanisms of degradation targeting specific organelles is vital for advancing our knowledge of plant development and stress responses. Novel research approaches are needed to elucidate the regulatory mechanisms of such disintegration and removal. An experimental design that combines ‘omics’ techniques with detailed ultrastructural observations will be instrumental in unraveling complexities beyond selective autophagy in organelle removal. Furthermore, advanced methods for such investigations already exist (Kułak et al. 2023), providing numerous opportunities to address many outstanding questions through studies of xylem and phloem differentiation. The combined use of various techniques enables the identification of organelles and even smaller cytoplasmic structures as well as their biogenesis or heterogeneity, exemplified by P-protein (Kulak et al. 2025). Other single-cell analyses of phloem development have revealed distinct cell states and their spatiotemporal dynamics. However, the study was supported by confocal long-term live imaging of sieve element–specific and nuclear-localized reporter lines (Roszak et al. 2021). The generation of lines with fluorescent protein-based organelle markers provides an excellent tool for tracking the trajectory of individual cytoplasmic elements (Wu et al. 2016). This method enables multiple transformations as well as the observation of interactions between different organelles and protein colocalization (Kriechbaumer and Brandizzi 2020; Nelson et al. 2007; Stellmach et al. 2022; Wang et al. 2023; Zhang et al. 2021). Moreover, organelle-specific fluorescent dyes can be added for such microscopic observations for plastids (Ichikawa et al. 2022), mitochondria (Zheng et al. 2009), vacuoles (Scheuring et al. 2015) and others (Schoor et al. 2015). Methods for fluorescent staining are also available for autophagosomes (Pu and Bassham 2016). High resolution detection of organelle removal pathways using fluorescent dyes or proteins is more challenging for inner tissues. It requires fixation and anatomical sectioning, which precludes the use of live-cell dyes. Furthermore, fluorescent proteins are also sensitive to fixation and embedding in resins or waxes. Nevertheless, several approaches have been developed to minimize the risk of signal loss (Pasternak et al. 2015) or to stain fixed tissues (Zelko et al. 2012). Low-temperature fixation and dehydration worked well in semithin and ultrathin sections in the case of labeling with fluorescent proteins in sieve elements (Bell et al. 2013).

Various modes of intracellular destruction against individual organelles were identified through ultrastructural studies in the twentieth century (see bibliography), yet many still remain mysterious at the molecular level. It is clear that upon developmental or environmental triggers, degradation is activated for cell destruction. However, the fate of each protoplast element remains unclear. The formation of transport conduits depends on such degradation. Hence, greater focus should be placed on the differentiation of vascular tissues, due to PCD mechanisms in xylem and the poorly understood PCD-like mechanisms in phloem. Advancing our understanding of organelle-specific degradation, through the integration of ultrastructural studies, live-cell imaging, and molecular approaches, will be crucial for deciphering how plants coordinate the development finalizing in death or specialization related to cytoplasmic reduction.

## Data Availability

No datasets were generated or analysed during the current study.
